# Psychometric properties of the Amharic version of the Infant-Toddler Home Observation for Measurement of the Environment (IT-HOME)

**DOI:** 10.1186/s40359-026-04350-7

**Published:** 2026-03-16

**Authors:** Anemut Mehari, Emebet Mulugeta

**Affiliations:** 1 School of Psychology, Addis Ababa University, Addis Ababa, Ethiopia; 2 Department of Psychology, Dilla University, Dilla, Ethiopia

**Keywords:** Validation, IT-HOME, Factor analysis, Measurement invariance, Ethiopia

## Abstract

Children’s future development is strongly shaped by their home environment. This includes their cognitive, social, and emotional growth. However, a culturally validated and reliable tool to measure the home environment in Ethiopia is still lacking. This study aims to validate the IT-HOME instrument for infants and toddlers from birth to 3 years. A total of 576 participants were recruited, of whom 458 completed the survey. The data randomly split into exploratory factor analysis (EFA; *n* = 229) and confirmatory factor analysis (CFA; *n* = 229) samples. Items were scored using a five-point Likert scale, replacing the original binary response format. Content validity was established through expert review, rating, and item refinement procedures. EFA supported a six-factor structure, which was subsequently confirmed by CFA with acceptable model fit indices (22 items, with 3–5 items per factor). Reliability analyses demonstrated strong internal consistency across all factors and the full scale, with McDonald’s ω values ranging from 0.70 to 0.87. Overall, the findings provide evidence consistent with the cultural relevance and sound psychometric properties of the adapted instrument for use with infants and toddlers, regardless of sex, and indicate its usefulness for practitioners and researchers in identifying risky home environments and informing tailored interventions.

**Supplementary Information:**

The online version contains supplementary material available at 10.1186/s40359-026-04350-7.

## Introduction

Research over the past three decades has consistently demonstrated that the home environment plays a fundamental role in infant and child development. Both the quality and quantity of the home environment including cognitive stimulation, emotional warmth, and structured routines are crucial for supporting children’s cognitive, emotional, and social development. These factors are particularly important in early childhood, a period during which foundational experiences shape long-term developmental outcomes. Through consistent interactions, meaningful stimulation, and supportive caregiving systems, the home environment facilitates critical aspects of developmental growth [1].

Although the home environment is pivotal, few tools assess caregiving in real home settings [2]. Among these, the Home Observation for Measurement of the Environment (HOME) [3] is a popular instrument [4] across diverse populations [5] for assessing the quality and quantity of the caregiving environment in which a child grows up. By measuring the levels of support, stimulation, and interaction a child receives at home, the HOME helps researchers and practitioners understand the environmental factors that shape development. The instrument evaluates the home experiences of infants, toddlers, and children, viewing them as active participants in their own development through interactions with objects, events, and family members [6].

Bettye Caldwell and her team developed the HOME during 1960s. Its original development was driven by the recognition that cognitive assessments alone cannot fully predict developmental outcomes. This recognition highlighted the need to understand how the environment affect cognitive development, the limitations of existing measures such as socioeconomic status, and the necessity of a comprehensive environmental assessment to inform intervention strategies [7]. The HOME profile draws on Bronfenbrenner’s [8] ecological systems theory, which conceptualizes child development as occurring within interconnected systems that exert varying influences. For example, the assessment focuses on the relationship between the child and the primary caregiver, a relationship that is central to the microsystem. This dyadic interaction forms the foundation for the development of broader interpersonal structures [6].

The HOME inventory remains a valuable assessment tool; however, its development dates back several decades. The most recent edition of the administration and scoring manual was published in 2001, and the item content has remained unchanged since that time [9]. As a result, contemporary validation is necessary to reflect substantial societal changes, including the diversification of family structures, evolving gender roles, and the increased sharing of childcare responsibilities. Norms surrounding child discipline have also shifted, and digital environments now play a central role in family life. When the HOME inventory was originally developed, single parenthood and diverse family forms were less prevalent [10], and caregiving responsibilities primarily fell to mothers [11]. Moreover, corporal punishment, once widely accepted, has now been prohibited in 63 countries [12]. Ethiopia also banned corporal punishment in Article 36(1) (e) of its 1995 Constitution [13] and joined the Global Partnership to End Violence against Children in 2021 [14]. The inventory also predates the widespread integration of digital environments, such as internet access and electronic devices, into everyday family routines. These technologies have become essential for information access and communication [15] and significantly shape home dynamics. The applicability of the HOME inventory may also be affected by broader societal changes, particularly in urban contexts characterized by rising unemployment, increasing rates of single parenthood, substance use, and exposure to violence [16]. Together, these shifts raise concerns about the continued relevance and effectiveness of the HOME inventory for assessing contemporary family environments [17]. Consequently, updated validation efforts are necessary to ensure the instrument remains appropriate and meaningful in today’s social context.

The measure has been translated into different languages and adjusted for various cultures, including Thai, Bangladesh, and India [18, 19]. The changes include adjusting the number of books required to meet specific goals, to better account for cultural differences in resource availability [20]. Despite its widespread use across diverse populations and disciplines and its ability to correlate infancy and childhood scores with later cognitive assessments, such as the Wechsler Intelligence Scale for Children (WISC) [21], the HOME inventory has several limitations. First, most studies linking HOME scores to later developmental outcomes rely on correlational designs, which limits causal inference. Second, the instrument’s binary scoring system (e.g., whether a behavior occurred at least once in the past week) simplifies administration but omits important qualitative and quantitative information, such as the frequency or intensity of specific caregiving behaviors (e.g., physical punishment). The HOME inventory also fails to capture variations in caregiving practices among siblings within the same household [4]. Additionally, the measure relies on data collected at a single time point, primarily reflecting caregiver behavior during the assessment period. This may introduce bias, as participants may modify their behavior to conform to socially desirable expectations or behave atypically during observation.

The instrument has not been thoroughly validated for assessing home environments in Ethiopia, which limits understanding of home environment profiles, hinders the development of evidence-based interventions to improve child development outcomes, and makes the application of its psychometric indices in this context methodologically unsound. Therefore, validating the instrument to capture key nurturing qualities across six subconstructs and to identify strengths and weaknesses in home environments is essential for its use in research and practice [6]. However, validation efforts remain confined to Ethiopian urban families.

Of the four HOME inventory versions designed for different developmental age groups [22], the infant–toddler version (IT-HOME), which assesses the quality of the home environment and caregiver–child interactions for children from birth to 3 years was validated. Because the home is a central ecological context where infants and toddlers spend approximately 90% of their time [23]. Its six subscales with its description and example items are shown in Table 1.

**Table 1:** Subscales, descriptions, and example items of the IT-HOME inventory

Subscale	Description	Example item
Responsivity	Child-caregiver emotional and verbal interactions	Parent responds verbally to child’s verbalizations
Learning materials	Presence of age-appropriate toys or learning materials	The child has access to eye-hand coordination toys
Involvement	Parental involvement/interaction with the child (physically)	Parent provides toys that challenge child to develop new skills
Restriction avoidance	Discipline is applied through restriction or punishment within an atmosphere of acceptance.	Parent does not shout at child
Environmental organization	Organizing the child’s time outside the family house and looks of the child’s personal space	Child’s play environment is safe
Stimulation variety	The child’s daily routine provides opportunities for stimulation and variety through social interactions beyond the primary caregiver.	Child has access to books of his or her own

The IT-HOME dimensions were derived and validated within a clearly articulated theoretical framework. The historical and theoretical literature on home learning environments and ecological systems perspectives informed the conceptual definition of each dimension. These frameworks emphasize the roles of material resources, caregiver–child interactions, and broader contextual supports in shaping developmental outcomes, which directly correspond to the constructs operationalized in the IT-HOME. These theoretically grounded dimensions were empirically tested to evaluate construct, factorial, and structural validity, examining whether the observed structure aligned with the hypothesized conceptual model. Convergence between theoretical expectations and empirical findings provided evidence supporting the validity and reinforced the conceptual foundation of the IT-HOME instrument within the Ethiopian urban family context. In light of the aforementioned limitations, the present study addresses them through:

- (i) cultural and linguistic adaptation;
- (ii) modernization of the response format by replacing the dichotomous (yes/no) scale with a five-point Likert scale ranging from “never exhibits the behavior” to “always exhibits the behavior,” thereby allowing for greater nuance and variability in responses; and
- (iii) structural validation including measurement invariance by infant’s and toddler’s sex.

## Methods

### Study setting

This study was carried out in Addis Ababa, Ethiopia’s capital, which encompasses individuals from a range of socioeconomic, religious, and ethnic backgrounds. Ethiopia is a low-income country with a per capita gross national income of $1,020. The country, located in East Africa, has a population exceeding 135.5 million [25], approximately 80% of whom live in rural areas with limited access to education and healthcare [26].

The capital comprises 11 subcities. In 2023, its population is estimated at 3.9 million [27], accounting for 23.20% of Ethiopia’s total urban population. The city’s subcities are classified into three groups - high, medium, and low - based on revenue-generating capacity and socioeconomic characteristics [28]. Considering this classification and Ethiopia’s label as a low-income country by the World Bank [29], the Gulele subcity was selected for the study. Located in the northern part of the capital, Gulele covers 3,119.09 hectares (31.19 km²), representing approximately 6% of the city’s total land area. Administratively, the it is divided into 10 districts (locally known as Woredas), which constitute the smallest units of administration [30]. This study sampled 50% of these districts (Districts 1, 3, 5, 7, and 9). The included districts reflect the sub-city’s geographic and administrative diversity. For instance, Districts 1 and 3 are located in the northern and central areas of the sub-city, District 5 comprises a mixed residential–commercial zone, and Districts 7 and 9 are situated in the southern and peripheral regions.

### Study design

Community-based cross-sectional survey design was used.

#### Participants and sampling

Participants were selected from the pediatric care units of five health centers using a stratified random sampling technique. Within each center, eligible study units were identified using computer-generated random numbers. An initial sample size of 384 participants was determined via Cochran’s [24] formula for estimating a representative sample of proportions, ${\mathrm{n}}_{0}=\frac{{\mathrm{z}}^{2}\mathrm{p}\mathrm{q}}{{\mathrm{e}}^{2}}$. To account for a large population with unknown variability, a population proportion (p) of 50% to maximize variability was assumed. A confidence level of 95% and a margin of error (e) at ± 5% were set [31]. Because the survey employed a complex stratified sampling design, a default design effect (Deff = 1.5) was adapted [32]. This increased the required sample size to 576 participants, with 115 participants selected from each of four districts and 116 participants from the remaining district, using an equal allocation across strata: ${\mathrm{s}\mathrm{a}\mathrm{m}\mathrm{p}\mathrm{l}\mathrm{e}\,\mathrm{s}\mathrm{i}\mathrm{z}\mathrm{e}\,\mathrm{f}\mathrm{o}\mathrm{r}\,\mathrm{d}\mathrm{i}\mathrm{s}\mathrm{t}\mathrm{r}\mathrm{i}\mathrm{c}\mathrm{t}\,(\mathrm{n}}_{\mathrm{i}})=\frac{\mathrm{t}\mathrm{o}\mathrm{t}\mathrm{a}\mathrm{l}\,\mathrm{s}\mathrm{a}\mathrm{m}\mathrm{p}\mathrm{l}\mathrm{e}\,\mathrm{s}\mathrm{i}\mathrm{z}\mathrm{e}\,\left(\mathrm{n}\right)}{\mathrm{n}\mathrm{u}\mathrm{m}\mathrm{b}\mathrm{e}\mathrm{r}\,\mathrm{o}\mathrm{f}\,\mathrm{d}\mathrm{i}\mathrm{s}\mathrm{t}\mathrm{r}\mathrm{i}\mathrm{c}\mathrm{t}\mathrm{s}\left(\,\mathrm{k}\right)}$. Of the 576 participants recruited, 458 completed the survey, yielding a response rate of 79.5%. To minimize non-response, up to two contact attempts were made on different days (one via phone call and one in person). The final number of participants was considered adequate, as it exceeded the commonly accepted minimum threshold of 70% for social science research [33]. Measures were taken to ensure the representativeness of the sample, and all procedures followed established methodological standards.

### Eligibility criteria

**Fig. 1:**
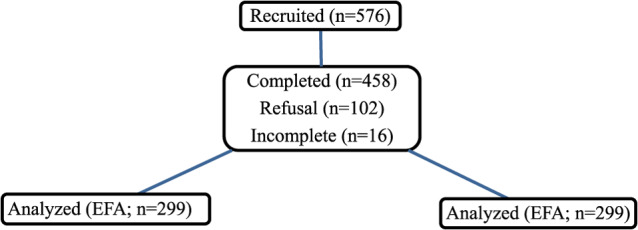
CONSORT-style flow diagram summarizing participant recruitment and retention for analysis

Participants were eligible if they (1) had resided in the selected district for at least six months, (2) had children aged ≤ 3 years, and (3) were able and willing to provide informed consent. Individuals were excluded if they were temporary residents or visitors, unable to provide informed consent, or submitted incomplete data. All eligible individuals recruited were approached and invited to participate (Fig. 1). Participation was voluntary, and individuals who declined were classified as refusals and were not replaced.

Factor analysis adequacy was assessed prior to item reduction. The sample size met commonly recommended guidelines, with a participant-to-item ratio of 10:1 [34, 35]. Sampling adequacy was further supported by a Kaiser–Meyer–Olkin (KMO) value of 0.78, indicating that the data were suitable for factor analysis. Based on evidence from simulation studies of factor solutions, moderate to high factor loadings (≥ 0.50) were assumed, as these allow stable and accurate estimation and can be detected reliably [36]. Finally, the single dataset was randomly split into two subsamples. This was done following the commonly recommended approach of allocating data approximately evenly between EFA and CFA when the overall sample size is sufficiently large [35], while preserving comparable distributions of participant characteristics across subsamples.

### Instrument

The instrument contains two primary sections: background information and the IT-HOME inventory. The IT-HOME inventory, adapted from Caldwell and Bradley [9] and designed for children aged birth to 3 years, includes 45 items organized into six subscales. The scoring format was changed from a binary response to a five-point Likert scale - “never” (1), “rarely” (2), “sometimes” (3), “often” (4), and “always” (5) - to capture greater variability in responses. Additionally, time-bound phrasing (e.g., “during the visit”) was removed so that items reflect general behaviors rather than those limited to a single observation period. All items were positively worded.

#### Two-stage instrument reviews

##### Qualitative review 

Initially, each item underwent a detailed, item-by-item analysis. A panel of three experts - one with a Ph.D. and two with M.A. degrees in Educational Measurement and Evaluation -were recruited and reviewed each item, discussed them, and provided feedback. This process involved modifying, revising, or rewording items to ensure cultural and contextual relevance as well as face validity. Sample reworded items include “the child has access to substitute care” instead of “substitute care is provided by one of three regular substitutes,” “child gets out of the house” instead of “child gets out of house at least four times/week (12 m),” and “child gets a special place for toys or play materials” instead of “child has a special place for toys and treasures.” Moreover, to consider cultural contexts, items such as “parent reads stories to child at least three times weekly” changed to “parent reads or tells stories (including religious, moral, and ethical scripts) to the child”. Temporal references, such as “during the visit,” were removed from item phrasing and revised to indicate whether the behavior occurred beyond what was directly observed. All conflicting views were resolved through discussion.

##### Quantitative review 

The items were also evaluated quantitatively via Lawshe’s [37] content validity ratio (CVR) method: CVR$=\frac{ne-\frac{N}{2}}{\frac{N}{2}}$. Where ne = the number of panelists pointing to the item as ‘essential’; N = the total number of panelists. Each item was rated on any of three rating scales: “essential”, “useful but not essential”, and “not essential”. This guides decisions about retaining or removing items from the initial pool. The overall content validity of the tool was computed using content validity index (CVI). This was done by finding the average of the CVR values for the chosen items [38]. The CVR value ranges from − 1 to 1. Positive values indicate that the item is acceptable and clear. Negative values indicate that the item needs revision, modification, or rejection. A CVR value of zero means that 50% of the subject matter experts (SMEs) in a panel of size N see the item as essential, deeming it valid.

Lawshe set rules for evaluating CVR values with different panel sizes using a 1-tailed test at the 0.05 significance level. Nevertheless, other researchers have used various panel sizes and produced effective CVR values. For example, studies had 12 evaluators (1) [39], 10 evaluators [40, 41], 9 evaluators [42], and 8 evaluators [43]. A panel of five evaluators—three doctoral students in Applied Developmental Psychology and two with M.A. degrees in Educational Psychology - was recruited and evaluated to meet Lawshe’s minimum requirement and has been shown to be effective, as demonstrated in the study by Mehari et al. [44].

The two items from the “restriction avoidance” subscale “at least 10 books are present and visible” and “family has a pet” were excluded due to both conceptual and empirical reasons. Content validity analysis showed negative CVRs for these items, indicating that experts did not consider them essential or relevant within the Ethiopian urban context. Conceptually, both items reflect aspects of material resources or environmental enrichment rather than caregiver behaviors related to limiting or avoiding restriction of the child. Therefore, the content does not align with the theoretical definition of the restriction avoidance subscale, which focuses on caregiver practices that allow autonomy and minimize unnecessary constraints. All other remaining items for *N* = 5 initially achieved a CVR of 1.0, which was adjusted to 0.99 to meet the acceptability threshold [45]. This adjustment accounts for the fact that items with unanimous expert agreement can produce a CVR of 1.0, which may overestimate content validity in small expert panels. To correct for this potential inflation, the Ayre and Scally's [46] adjustment was applied, yielding slightly lower CVR values that account for chance agreement and panel size, providing a more conservative and reliable estimate of content validity. After this procedure, all retained items reached an adjusted CVR of 0.99, demonstrating strong content validity (Annex I).

#### Instrument translation

The instrument was translated into Amharic, the official language of the study area. Three bilingual experts holding M.A. degrees in English language and literature and native in Amharic were recruited and conducted the translation process. One expert performed the forward translation into Amharic, another carried out the back-translation into English, and the third expert, who has also a background in psychology, reviewed both versions to verify conceptual and linguistic equivalence between the original and translated instruments.

A single-phase cognitive interview was conducted with *n* = 5 participants to assess item clarity and comprehension [47]. This approach involved in-depth interviews to examine how the target audience interpreted and responded to the items [48]. Participants suggested minor wording changes to better reflect their interpretations, and discrepancies were resolved by revising item wording to improve clarity while preserving the original construct meaning [45].

### Data collection procedure

Data were collected between February and April 2025. Caregivers of children from birth to 3 years, corresponding to the randomly selected study units, were contacted at health centers, where they receive vaccination and other infant health services. The researcher, along with assistants, visited the homes of parents who agreed to help with the data collection. Five experienced health extension professionals were recruited and trained as data enumerators. Data were collected from the primary caregiver, typically the mother, in the home setting. The scoring method was revised from a dichotomous yes/no format to a five-point frequency-based Likert scale, integrating both observation and interview items. This approach allowed assessors to capture the home environment more accurately by considering both direct observations and caregiver reports. For example, play materials that are not consistently available at home may still be accessible to children at varying frequencies through public play spaces. During the assessment, the child interacted with both the caregiver and the data collector. Although data were collected using two modalities - paper-based and the web-based Kobo Toolbox platform - no mode analysis was conducted to assess its potential effects on factor loadings or measurement invariance. The assessment procedure took approximately 30–45 minutes.

### Data analysis

Key data assumptions were checked prior to factor analysis. Validity and underlying factor structures were explored. Internal consistency was examined using reliability and/or composite reliability tests. Convergent validity was tested via average variance extracted (AVE) values. Then, divergent validity was explored using the Fornell–Larcker test. Underlying factor structures were confirmed through factor analysis. Configural, metric, and scalar invariance models were used to check measurement invariance across children’s sex.

Prior to conducting the data analysis, the data entry correctness and suitability for further analysis were checked. Multivariate outliers were identified using Mahalanobis distance, and one case exceeding the critical χ² value at *p* < 0.001 was removed prior to analysis. Sensitivity analyses showed that this removal did not affect the results or model fit. The Kolmogorov‒Smirnov test (0.04 (229), *p* = 0.20) and the Shapiro‒Wilk test (W (229) = 0.99, *p* = 0.05) confirmed that the score distributions were normal. The skewness value was -0.24, with a standard error of 0.16. The kurtosis value was -0.27, with a standard error of 0.32. This shows that the data are slightly skewed to the left but close to zero. It falls within the standard normal distribution range (Z ± 1.96), suggesting a normal distribution of the data [49]. Overall, because the Shapiro–Wilk test was borderline (*p* = 0.05) and the items were ordinal Likert-type, maximum likelihood estimation with robust corrections (MLR) was applied to account for marginal non-normality and the ordinal nature of the data.

## Results

### Participants' characteristics

The largest group at the visit were mothers, accounting for 38% of the participants. The average age of the participants was 34 years. Caregivers had different levels of education. One-fourth of the mothers (25.30%) had a diploma. In contrast, one-third of fathers (34.10%) held a first-degree qualification. Compared with mothers (49.80%), more fathers (93.90%) were engaged in work, either hired or self-employed.

Households had an average size of five members, with a mean monthly income of 19,459.83 Birr. Among the children included, slightly over one-third (32.3%) were firstborn, with a mean age of 2 years. Most participants (72.10%) had a current care arrangement. Additionally, over half (62%) were female, a share that may reflect the expected sex distribution in the population (Table 2).

**Table 2:** Demographic characteristics of the study participants (*n* = 458)

Demographic variables	*N*	Percentage
Participant present during visit		
Mother	174	38
Father	130	28.40
Sister	70	15.30
Caregiver	42	9.20
Aunt	42	9.20
Maternal/caregiver age		
Mean (Sd)	34.16 (6.57)	
Max.	45	
Min.	19	
Mother education level		34.50
1^st^ degree	158	12.70
2^nd^ degree and above	58	25.30
Diploma	116	15.30
High school	70	12.20
Elementary	56	
Father education level		
1^st^ degree	156	34.10
2^nd^ degree and above	120	26.20
Diploma	98	21.40
High school	56	12.20
Elementary	28	6.10
Is the mother employed?		
Yes	228	49.80
No	230	50.20
Is the father employed?		
Yes	430	93.90
No	28	6.10
Monthly income level (in birr)		
Mean (Sd)	19459.83(18250.20)	
Median (IQR)	15,000(10000)	
Family size		
Mean (Sd)	5 (2)	
Max.	9	
Min.	1	
Child sex		
Male	174	38
Female	284	62
Child birth order		
1^st^	148	32.30
2^nd^	132	28.80
3^rd^	54	11.80
4^th^	68	14.80
5^th^	42	9.20
6^th^	14	3.10
Child age		
Mean (Sd)	2.29 (0.76)	
Max.	3	
Min.	1	
Current child care arrangements		
Yes	330	72.10
No	128	27.90

### Validity analysis

#### Factorial validity

##### Exploratory factor analysis 

Half of the data (*n* = 229) were subjected to EFA. Multiple criteria were used to retain factors. These include eigenvalues greater than one [50]; visual inspection of Cattell’s [51] scree plot; parallel analysis [52] comparing observed eigenvalues with those from randomly generated datasets and retaining only factors exceeding the corresponding random values; Bartlett’s [53] chi-square test; and exclusion of factors containing fewer than three items [35].

**Fig. 2:**
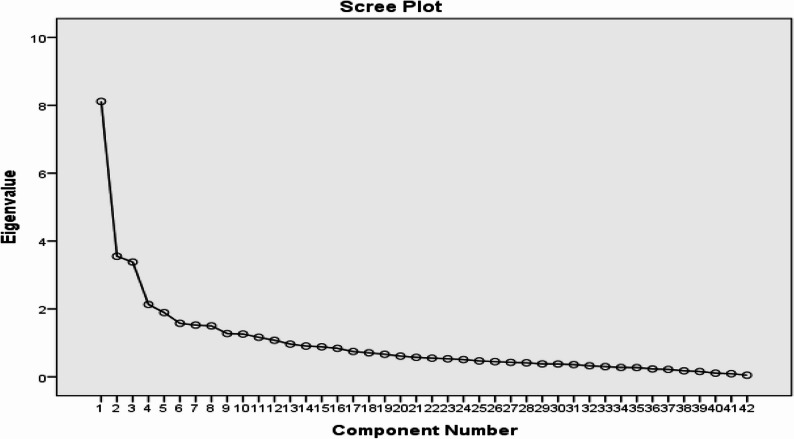
Scree plot

**Fig. 3:**
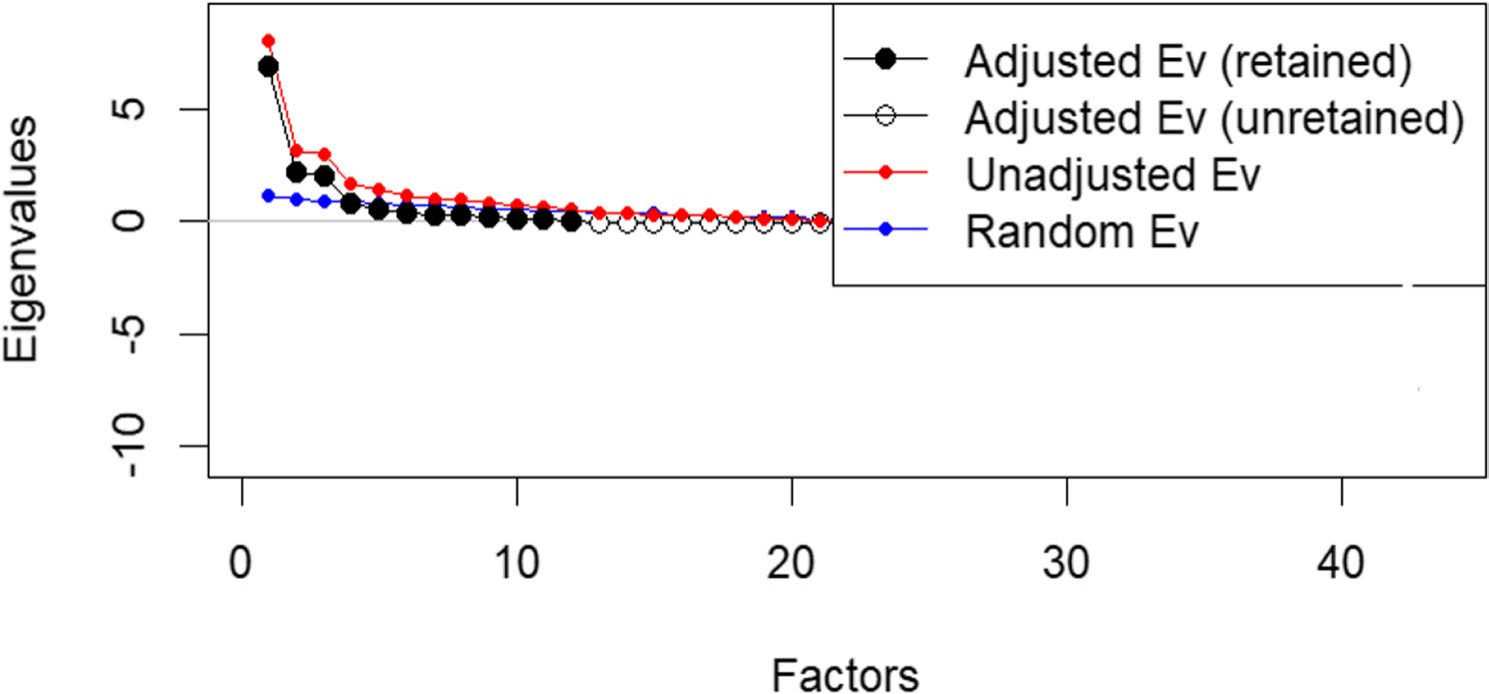
Parallel analysis

Accordingly, using principal axis factoring (PAF) with Promax oblique rotation, the KMO measure of sampling adequacy was greater than 0.60 and Bartlett’s test of sphericity yielded a value of 4706.02 (*p* = 0.00). Twelve factors were extracted using an item factor loading of ≥ 0.50. These factors had eigenvalues of ≥ 1 and explained 67.73% of the variance (Table 3). The scree plot in Fig. 2 and the parallel analysis (based on the R program) in Fig. 3 show that 12 factors should be retained. Finally, six factors comprising 28 items were extracted, each meeting the criterion of at least three items per factor (Table 4). Together, these factors explained 61.56% of the total variance, exceeding the minimum acceptable threshold of 50% and indicating that the factor structure has adequate explanatory power [54].

**Table 3:** Total variance explained

Component	Initial Eigenvalues			Extraction Sums of Squared Loadings			Rotation Sums of Squared Loadings		
	Total	% of Variance	Cumulative %	Total	% of Variance	Cumulative %	Total	% of Variance	Cumulative %
1	8.12	19.32	19.32	8.12	19.32	19.32	4.53	10.78	10.78
2	3.55	8.46	27.78	3.55	8.46	27.78	3.59	8.56	19.33
3	3.38	8.05	35.83	3.38	8.05	35.83	3.37	8.03	27.36
4	2.13	5.07	40.90	2.13	5.07	40.90	2.79	6.64	34.01
5	1.89	4.50	45.41	1.89	4.50	45.41	2.70	6.43	40.44
6	1.58	3.75	49.16	1.58	3.75	49.16	2.03	4.84	45.28
7	1.52	3.63	52.79	1.52	3.63	52.79	1.86	4.44	49.72
8	1.50	3.57	56.36	1.50	3.57	56.36	1.79	4.26	53.98
9	1.27	3.03	59.39	1.27	3.03	59.30	1.65	3.92	57.89
10	1.26	3.00	62.39	1.26	3.00	62.39	1.60	3.80	61.69
11	1.17	2.78	65.17	1.17	2.78	65.17	1.29	3.07	64.76
12	1.08	2.56	67.73	1.08	2.56	67.73	1.25	2.97	67.73

Items with cross-loadings ≥ 0.30 [55] were removed, and the proportion of variance in each observed variable explained by the extracted factors was also adequate, with all communalities ≥ 0.30 [56]. Finally, the six-factor solution aligns with the theoretical development of the instrument, which originally identified and validated six as distinct factors [3, 9, 22].

**Table 4:** Rotated component matrix

Items	1	2	3	4	5	6	Communalities
Parent spontaneously vocalizes to the child	0.85						0.96
Parent responds verbally to child’s verbalizations	0.83						0.81
Parent tells child name of object/person in “teaching style”	0.76						0.57
Parent’s speech is distinct and audible	0.70						0.67
Parent spontaneously praises child	0.70						0.51
Parent caresses/kisses/hugs child	0.69						0.56
Child has access has to muscle activity toys or equipment		0.74					0.55
Child has access to stroller or walker, kiddie car, scooter, or tricycle		0.74					0.57
Child has access to learning equipment appropriate to age (cuddly or role-playing toys)		0.67					0.63
Child has access to learning facilitators—mobile table and chairs, high chair, play pen, toys for literature and music		0.57					0.53
Child has access to eye-hand coordination toys		0.53					0.47
Parent keeps child in visual range, looks at often			0.85				0.78
Parent talks to child while doing household work			0.83				0.40
Parent invests maturing toys with value via personal attention			0.76				0.40
Parent structures child’s play periods			0.59				0.66
Parent provides toys that challenge child to develop new skills			0.57				0.77
Parent does not shout at child				0.80			0.62
Parent does not express annoyance with or hostility to child				0.78			0.30
Parent does not scold or criticize child				0.77			0.74
Parent does not interfere or restrict child				0.57			0.77
Child has access to substitute care					0.81		0.57
Child gets out of house					0.78		0.68
Child gets a special place for toys or play materials					0.63		0.48
Child’s play environment is safe					0.51		0.44
Parent reads or tells stories (including religious, moral, and ethical scripts) to the child						0.67	0.57
Child eats with mother and father						0.61	0.30
Family visits with relatives or friends						0.61	0.62
Child has access to books of his or her own						0.52	0.63

*1* Responsivity, *2* Learning materials, *3* Involvement, *4* Restriction avoidance, *5* Environmental organizations, *6* Stimulation varieties

##### Confirmatory factor analysis 

**Fig. 4:**
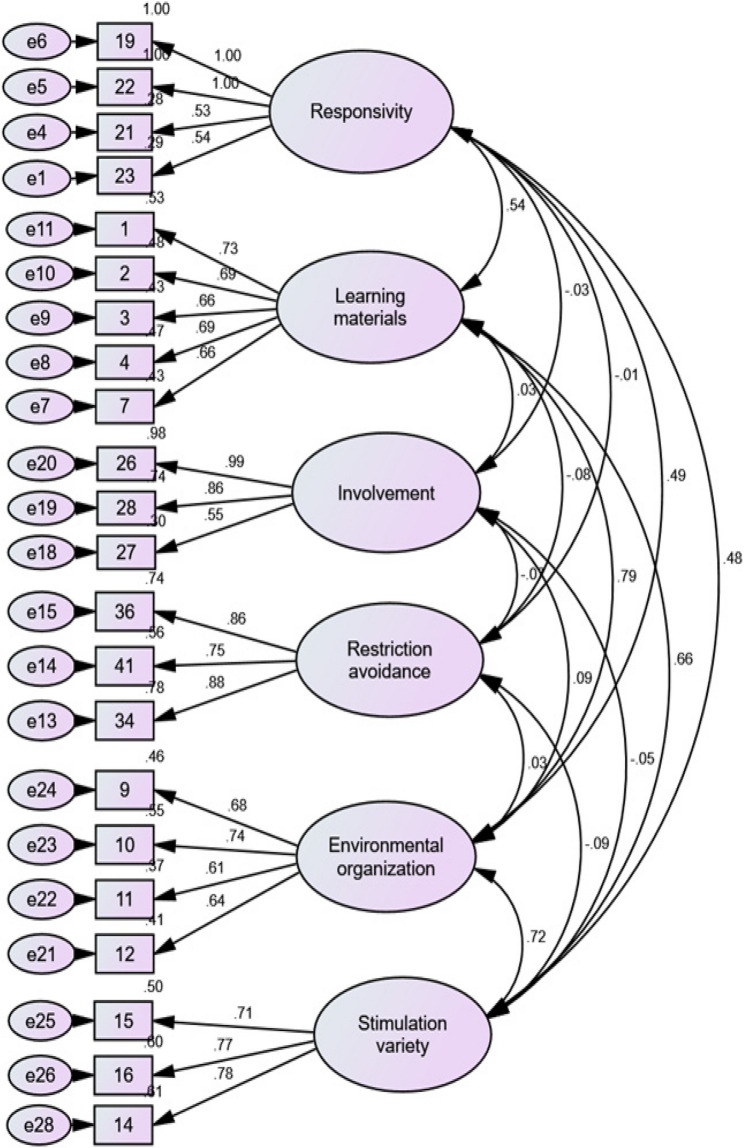
Six-factor measurement model

CFA was performed on the remaining half of the dataset (*n* = 229). Items with factor loadings below 0.50 were excluded. Fig. 4 shows that the base model fit indices were χ² = 471.12, df = 215; CMIN/DF = 2.19; CFI = 0.93; RMSEA = 0.07, 95% CI [0.06, 0.08]; and TLI = 0.91, indicating that the model met commonly recommended fit criteria (CMIN/DF < 5, CFI and TLI > 0.90, RMSEA < 0.08). However, GFI = 0.84, AGFI = 0.80, NFI = 0.87, and SRMR = 0.08 suggest that further improvement of model fit may be warranted [57]. Based on both conceptual and methodological considerations, Item 24 was removed from the model. Standardized residual covariances were examined to identify problematic items, and conceptual evaluation indicated that Item 24 (Parent’s speech is distinct and audible) was weakly aligned with the construct. Unlike the remaining indicators, which capture interactive, responsive, and affective parental behaviors, Item 24 reflects a speech clarity or delivery characteristic rather than a relational or interactional behavior. Its removal improved model fit and enhanced the conceptual coherence of the construct. The model demonstrated good fit with the following indices: χ² = 340.34, df = 194; CMIN/DF = 1.75; CFI = 0.96; TLI = 0.95; RMSEA = 0.06, 95% CI [0.04, 0.07]; SRMR = 0.07; GFI = 0.96; AGFI = 0.90; and NFI = 0.90 (Table 5).

**Table 5:** Model fit indices

Improving model	Fit indices	CMIN/DF < 5	≥ 0.9					≤ 0.08	
			GFI	AGFI	CFI	NF I	TLI	SRMR	RMSEA
Base	*χ2* = 471.12, *df* = 215, *p* = 0.00	2.19	0.84	0.80	0.93	0.87	0.91	0.08	0.07
Removal of item 24	*χ2* = 340.34, *df* = 194, *p* = 0.00	1.75	0.90	0.90	0.96	0.90	0.95	0.07	0.06

*χ2* chi-square, *df* degree of freedom, *CMIN* chi-square minimum discrepancy, *GFI* goodness-of fit index, *AGFI* adjusted goodness-of-fit index, *CFI* comparative fit index, *NFI* normed fit index, *TLI* Tucker‒Lewis index, *SRMR* standardized root mean square residual, *RMSEA* root mean square error approximation

##### Measurement invariance 

A multigroup confirmatory factor analysis (MGCFA) with 174 male and 284 female participants was conducted to examine whether the inventory functioned equivalently across sexes. Measurement invariance (MI) was tested sequentially using three increasingly restrictive models: configural, metric, and scalar invariance. First, configural invariance was evaluated to determine whether the factor structure and pattern of item loadings were consistent across sex groups. The configural model demonstrated acceptable fit, as indicated by overall model fit indices (CFI ≥ 0.90; RMSEA ≤ 0.08). Next, metric invariance was tested to assess the equivalence of factor loadings across groups, followed by scalar invariance to evaluate the equality of item intercepts. MI was supported at multiple levels. The configural model showed acceptable fit (χ² = 506.54, df = 161; CFI = 0.90; RMSEA = 0.08, 95% CI [0.03, 0.07]). Metric invariance was also supported (χ² = 601.40, df = 190), with no meaningful change in model fit relative to the configural model (ΔCFI = 0.00; ΔRMSEA = 0.00, 95% CI [0.01, 0.06]). Similarly, scalar invariance was established (χ² = 670.10, df = 210), again showing negligible changes in fit (ΔCFI = 0.00; ΔRMSEA = 0.00, 95% CI [0.01, 0.05]). All models met established equivalence criteria (ΔCFI ≤ 0.01; ΔRMSEA ≤ 0.02; [58]), as summarized in Table 6.

**Table 6:** Measurement invariance across sex groups

Model	χ2	df	RMSEA (95% CI)	CMIN/DF	CFI	∆CFI	∆RMSEA	Decision
CI	506.54	161	0.07 (0.03, 0.07)	3.14	0.90			
MI	601.40	190	0.07 (0.01, 0.06)	3.16	0.90	0.00	0.00	Equivalence
SI	670.10	210	0.07 (0.01, 0.05)	3.19	0.90	0.00	0.00	Equivalence

*CI* Configural invariance, *MI* metric invariance, *SI* scalar invariance, *CMIN* chi-square minimum discrepancy, *df* degree of freedom, *CFI* comparative fit index, *RMSEA* root mean square error approximation, *ΔCFI* change in values of CFI, *ΔRMSEA* change in values of RMSEA

The tool demonstrated equivalence across three key areas: factor structure, factor loadings, and item intercepts. This indicates that parents of both male and female infants and toddlers interpret the items invariantly. Overall, of the 43 initially administered items, 28 were retained after EFA, and 22 remained in the final CFA, demonstrating measurement invariance across the sex of the infants and toddlers.

#### Construct validity

Two components of construct validity were tested: convergent validity (CV) and divergent validity (DV). The average variance extracted (AVE) values ≥ 0.50 in Table 7 demonstrated the presence of CVs. Subscales, including learning materials and environmental organization, had an AVE of 0.50, meeting the minimum threshold for CV, though this indicates potential room for improvement. Future refinement could involve reviewing items for clarity, relevance, and consistency, and revising or replacing items with lower factor loadings to strengthen the subscales’ convergent validity.

**Table 7:** Construct validity

No.	Subscales	AVE	Fornell-and-Larcker test					
			1	2	3	4	5	6
1	Responsivity	0.64	**0.80**					
2	Learning materials	0.50	0.54	**0.71**				
3	Involvement	0.68	-0.03	0.03	**0.82**			
4	Restriction avoidance	0.69	-0.01	-0.08	-0.07	**0.83**		
5	Environmental organization	0.50	0.49	0.68	0.09	0.03	**0.70**	
6	Stimulation variety	0.57	0.48	0.66	-0.05	-0.09	0.62	**0.80**

*AVE* Average Variance Extracted

*Bold values on the diagonal Square root of the AVE for each corresponding latent construct*

The DV was also achieved via the Fornell and Larcker [59] test. The square root of the AVE for each latent construct was greater than its correlations with other constructs (Table 6). This demonstrated the presence of DV [60]. The heterotrait–monotrait (HTMT) ratio of correlations between learning materials and environmental organization was 0.80, which is below the recommended thresholds of 0.85 [58] or 0.90 [61], further confirming the presence of DV.

### Reliability analysis

Table 8 shows that the reliability coefficients for the original IT-HOME subscales ranged from moderate (0.68) to strong (0.89) [6]. Its overall reliability was robust (0.80) [62]. The reliability coefficients in the EFA ranged from α = 0.68–0.89 and showed improvement in the CFA, with α values between 0.76 and 0.88. Moreover, coefficient omega, which provides an unbiased estimate for congeneric items [63, 64] was calculated. McDonald’s ω values ranged from 0.70 to 0.87, with ω = 0.86 for females and ω = 0.85 for males, all exceeding the recommended threshold of ≥ 0.70 [60]. These results indicate adequate reliability for the measured constructs. Factor loadings were also within the acceptable range (0.53–0.99; Annex II). Overall, the inventory demonstrates strong internal consistency and composite reliability (CR), supporting its psychometric soundness for assessing the home environment of infants and toddlers.

**Table 8:** Number of items with reliability indices

No.	Subscales	Items in the original scale	α	Expert review	EFA		CFA			
					Items after EFA	α	Items after CFA	α	CR	McDonald’s ω
1	Responsivity	11	0.68 to 0.89	11	6	0.89	4	0.86	0.90	0.87
2	Learning materials	9		9	5	0.81	5	0.82	0.82	0.81
3	Involvement	6		6	4	0.81	3	0.83	0.90	0.85
4	Restriction avoidance	8		6	4	0.78	3	0.88	0.87	0.87
5	Environmental organization	6		6	4	0.79	4	0.76	0.76	0.76
6	Stimulation variety	5		5	4	0.68	3	0.80	0.80	0.80
	IT-HOME	45	0.80	43	28	0.86	22	0.85		0.70

*α* Cronbach’s alpha, *CR* Composite reliability

### Dimension correlations and item‒total correlations

The item-total correlation helps us to determine whether individual items relate well to the full scale. The result shows a statistically significant correlation with a range of *r* (227) = 0.12–0.81, *p* ≤ 0.05. In addition, the components of the scale had a significant relationship with the full scale, as indicated by a range of *r* (227) = 0.20–0.81, *p* ≤ 0.01.

## Discussion

This study not only established the psychometric characteristics of the Amharic IT-HOME scale for infants and toddlers aged 0–3 years but also illuminated how home environmental characteristics in Ethiopia intersect with key developmental and ecological theories. The six-factor structure identified - responsivity, provision of learning materials, parental involvement, avoidance of punishment, organization of the environment, and variety stimulation - aligns with the classic IT-HOME dimensions while also reflecting culturally specific caregiving practices and broader socioecological contexts. Bronfenbrenner’s ecological model underscores that development occurs through dynamic interactions within and across systems, from immediate caregiver–child exchanges (microsystem) to broader cultural norms (macrosystem) that influence parenting practices [8]. This framework helps interpret why responsive interaction and structured stimulation emerged as distinct, meaningful dimensions: they represent proximal processes considered central to early development (e.g., supportive verbal engagement and emotional warmth predict cognitive and self-regulatory outcomes) in longitudinal and large-scale studies of home environments [65].

A structural comparison of the Amharic IT-HOME with recent international adaptations suggests both shared and unique features. For example, the HOME-21 revision for the 21st century identified links between enriched home environments and parental education, positive parenting practices, and household stability [66]. Similarly, our factor of parental involvement in the Ethiopian context captures active engagement in teaching, play, and routines that align with these broader constructs yet anchor them in locally salient caregiving patterns, such as shared caregiving among extended family members. Additionally, cross-cultural invariance research across diverse low- and middle-income settings, such as the MAL-ED study, confirms that core HOME constructs can maintain measurement equivalence even when adapted for different environments, strengthening the case for the IT-HOME’s cross-context applicability [67].

Linking empirical findings to developmental theory, the emergence of verbal and emotional responsivity and opportunities for variety as distinct factors reflects how proximal caregiver interactions and environmental stimulation predict self-regulation and cognitive outcomes in early childhood [68]. These associations are consistent with contemporary evidence that quality home experiences—characterized by rich language, emotional support, and cognitively stimulating materials and activities—are associated with better socioemotional and cognitive outcomes [66].

Our focus on a 22-item final scale represented a deliberate balance between practical feasibility and conceptual coverage. While the original inventory included 45 items, paring down to 22 preserved coverage of six theoretically meaningful constructs without burdening respondents—an important trade-off for use in low-resource and large-scale field settings. This aligns with trends in the field toward shorter measures that reduce respondent fatigue while retaining psychometric rigor [65].

Measurement invariance analyses confirmed that the IT-HOME functions equivalently across male and female infants and toddlers, providing evidence that caregivers interpret and respond to items similarly regardless of child sex. This finding is essential for both research and program evaluation, supporting valid comparisons across groups and reflecting theoretical expectations that, although ecological circumstances vary, core developmental processes operate consistently in early childhood.

Finally, interpreting the IT-HOME dimensions within the Ethiopian context highlights several culturally specific patterns. For example, storytelling and shared verbal engagement are common caregiving practices that underpin the verbal and emotional responsivity factor; shared caregiving by extended family members reinforces parental involvement; and daily routines shaped by communal norms contribute to the organization of physical and temporal environments. In domains such as avoidance of punitive practices, the instrument captures home experiences in a context where formal policy moves toward prohibition of corporal punishment are still nascent, reflecting both emergent social change and cultural caregiving norms [13, 14]. In sum, the validated six-factor, 22-item Amharic IT-HOME not only holds strong internal consistency and construct validity but also extends the international literature on home environment measurement by offering a culturally grounded tool that aligns with contemporary theoretical frameworks and recent psychometric advances. Its alignment with both classic HOME constructs and modern adaptations (e.g., HOME-21) enhances its utility for research and interventions aimed at supporting child development in diverse contexts.

## Conclusion

In conclusion, the Amharic version of the IT-HOME tool demonstrates strong reliability and validity for assessing the home environment of infants and toddlers in urban contexts. Its manageable number of items makes it a practical and cost-effective instrument for use in resource-limited settings.

The tool provides valuable evidence for researchers examining the relationship between home environments and early child development, while also offering actionable insights for policy and practice. By identifying home environments with limited cognitive or emotional stimulation, IT-HOME can inform the design of targeted interventions, including caregiver training, provision of learning materials, and early childhood support programs, and can be applied in practice as a screening and program evaluation tool to monitor change and guide service delivery. Policymakers may use aggregated findings to prioritize resources for high-risk communities and inform urban child development policies and standards. Additionally, the tool supports practitioners in monitoring changes in the home environment over time, enabling evidence-based program adjustments and early referral to health, nutrition, and psychosocial services. Future research should examine its longitudinal predictive validity, extend validation to rural and socioeconomically diverse populations, and use uniform data collection modes to ensure the generalizability and robustness of the factor structure.

### Limitations

This study has several limitations. First, recruitment from a single urban sub-city involved in health facilities may limit the applicability of the findings to rural areas, other regions, and families not engaged with health services. Further validation is required to establish generalizability across diverse urban and rural settings. Second, the cross-sectional design restricts interpretation to concurrent associations and does not allow assessment of predictive validity or causal relationships. Third, data were collected using different administration modes (paper-based and Kobo Toolbox), and potential mode effects were not formally tested, which may have introduced measurement variability. Fourth, data collection during home visits may have introduced observer expectancy effects or social desirability bias, potentially influencing caregiver responses, observed behaviors, or evaluators’ assessments. Despite efforts to minimize these biases (e.g., standardized protocols and anonymity assurances), they cannot be fully ruled out. Fifth, test–retest reliability was not assessed, limiting the ability to verify the stability of the instrument over time. Sixth, due to the lack of testing for criterion validity, the tool’s validity relative to other developmental or environmental measures is unknown. Seven, measurement invariance was not established across key subgroups (caregiver education and sex, and child age and birth order), which may limit the comparability of scores across these groups. All of the above limitations may have influenced the observed factor structure and measurement invariance.

## Supplementary Information

Supplementary Material 1.

Supplementary Material 2.

## Data Availability

The data are available from the corresponding author upon reasonable request.

## Abbreviations

HOME: Home Observation for the Measurement of the Environment
EFA and CFA: Exploratory Factor Analysis and Confirmatory Factor Analysis
