# Supplementary material for: Psychometric properties of the Amharic version of the Infant-Toddler Home Observation for Measurement of the Environment (IT-HOME)
Source: BMC Psychol. 2026 Mar 16;14:592. doi: 10.1186/s40359-026-04350-7 (PMC13104347; doi:10.1186/s40359-026-04350-7)
Supplement: Supplementary file 1 — Supplementary Material 1. [file 40359_2026_4350_MOESM1_ESM.docx]

**Annex III: CFA items with factor loadings**

| S/N | **Factors** | **Item no.** | **Items** | **Estimate** | **Mean** | **Sd** |
| --- | --- | --- | --- | --- | --- | --- |
| 1 | Responsivity | Q23 | Parent caresses/kisses/hugs child | .543 | 13.19 | 2.92 |
|  |  | Q21 | Parent tells child name of object/person during visit in “teaching style” | .528 |  |  |
|  |  | Q22 | Parent responds verbally to child’s verbalizations | .998 |  |  |
|  |  | Q19 | Parent spontaneously vocalizes to the child | .999 |  |  |
| 2 | Learning materials | Q7 | The child has access to learning facilitators—mobile table and chairs, high chair, play pen, toys for literature and music | .658 | 16.09 | 3.55 |
|  |  | Q4 | The child has access to learning equipment appropriate to age (cuddly or role-playing toys) | .686 |  |  |
|  |  | Q3 | The child has access to eye-hand coordination toys | .657 |  |  |
|  |  | Q2 | The child access has to muscle activity toys or equipment | .694 |  |  |
|  |  | Q1 | The child has access to stroller or walker, kiddie car, scooter, or tricycle available | .726 |  |  |
| 3 | Restriction avoidance | Q34 | Parent does not scold or criticize child | .881 | 11.21 | 2.77 |
|  |  | Q41 | Parent does not shout at child during visit | .748 |  |  |
|  |  | Q36 | Parent does not interfere or restrict child | .860 |  |  |
| 4 | Involvement | Q27 | Parent structures child’s play periods | .549 | 10.64 | 2.57 |
|  |  | Q28 | Parent provides toys that challenge child to develop new skills | .862 |  |  |
|  |  | Q26 | Parent keeps child in visual range, looks at often | .992 |  |  |
| 5 | Environmental organization | Q12 | Child’s play environment is safe | .640 | 12.87 | 2.91 |
|  |  | Q11 | The child has access to substitute care | .606 |  |  |
|  |  | Q10 | Child gets out of house | .743 |  |  |
|  |  | Q9 | Child gets a special place for toys or play materials | .680 |  |  |
| 6 | Stimulation variety | Q14 | Child has access to books of his or her own | .780 | 9.79 | 2.46 |
|  |  | Q16 | Family visits with relatives or friends | .772 |  |  |
|  |  | Q15 | Parent reads stories (including religious readings) to child | .710 |  |  |
| IT-HOME | | | | | 73.79 | 10.38 |
