# Supplementary material for: Psychometric properties of the Amharic version of the Infant-Toddler Home Observation for Measurement of the Environment (IT-HOME)
Source: BMC Psychol. 2026 Mar 16;14:592. doi: 10.1186/s40359-026-04350-7 (PMC13104347; doi:10.1186/s40359-026-04350-7)
Supplement: Supplementary file 2 — Supplementary Material 2. [file 40359_2026_4350_MOESM2_ESM.docx]

**Annex I** Expert Content Validity Evaluation the IT-HOME inventory

| **Items numbers** | **Panelists** | | | | | | | | |
| --- | --- | --- | --- | --- | --- | --- | --- | --- | --- |
|  | **1** | **2** | **3** | **4** | **5** | **Experts in agreement** | **CVR _critical_** | **CVR adjusted** | **Decision** |
| 1 | 3 | 3 | 3 | 3 | 3 | 5 | 1 | 0.99 | Retained |
| 2 | 3 | 3 | 3 | 3 | 3 | 5 | 1 | 0.99 | Retained |
| 3 | 3 | 3 | 3 | 3 | 3 | 5 | 1 | 0.99 | Retained |
| 4 | 3 | 3 | 3 | 3 | 3 | 5 | 1 | 0.99 | Retained |
| 5 | 3 | 3 | 3 | 3 | 3 | 5 | 1 | 0.99 | Retained |
| 6 | 3 | 3 | 3 | 3 | 3 | 5 | 1 | 0.99 | Retained |
| 7 | 3 | 3 | 3 | 3 | 3 | 5 | 1 | 0.99 | Retained |
| 8 | 3 | 3 | 3 | 3 | 3 | 5 | 1 | 0.99 | Retained |
| 9 | 3 | 3 | 3 | 3 | 3 | 5 | 1 | 0.99 | Retained |
| 10 | 3 | 3 | 3 | 3 | 3 | 5 | 1 | 0.99 | Retained |
| 11 | 3 | 3 | 3 | 3 | 3 | 5 | 1 | 0.99 | Retained |
| 12 | 3 | 3 | 3 | 3 | 3 | 5 | 1 | 0.99 | Retained |
| 13 | 3 | 3 | 3 | 3 | 3 | 5 | 1 | 0.99 | Retained |
| 14 | 3 | 3 | 3 | 3 | 3 | 5 | 1 | 0.99 | Retained |
| 15 | 3 | 3 | 3 | 3 | 3 | 5 | 1 | 0.99 | Retained |
| 16 | 3 | 3 | 3 | 3 | 3 | 5 | 1 | 0.99 | Retained |
| 17 | 1 | 1 | 1 | 1 | 1 | 0 | -1 | 0.99 | Discarded |
| 18 | 1 | 1 | 1 | 1 | 1 | 0 | -1 | 0.99 | Discarded |
| 19 | 3 | 3 | 3 | 3 | 3 | 5 | 1 | 0.99 | Retained |
| 20 | 3 | 3 | 3 | 3 | 3 | 1 | 1 | 0.99 | Retained |
| 21 | 3 | 3 | 3 | 3 | 3 | 5 | 1 | 0.99 | Retained |
| 22 | 3 | 3 | 3 | 3 | 3 | 5 | 1 | 0.99 | Retained |
| 23 | 3 | 3 | 3 | 3 | 3 | 5 | 1 | 0.99 | Retained |
| 24 | 3 | 3 | 3 | 3 | 3 | 5 | 1 | 0.99 | Retained |
| 25 | 3 | 3 | 3 | 3 | 3 | 5 | 1 | 0.99 | Retained |
| 26 | 3 | 3 | 3 | 3 | 3 | 5 | 1 | 0.99 | Retained |
| 27 | 3 | 3 | 3 | 3 | 3 | 5 | 1 | 0.99 | Retained |
| 28 | 3 | 3 | 3 | 3 | 3 | 5 | 1 | 0.99 | Retained |
| 29 | 3 | 3 | 3 | 3 | 3 | 5 | 1 | 0.99 | Retained |
| 30 | 3 | 3 | 3 | 3 | 3 | 5 | 1 | 0.99 | Retained |
| 31 | 3 | 3 | 3 | 3 | 3 | 5 | 1 | 0.99 | Retained |
| 32 | 3 | 3 | 3 | 3 | 3 | 5 | 1 | 0.99 | Retained |
| 33 | 3 | 3 | 3 | 3 | 3 | 5 | 1 | 0.99 | Retained |
| 34 | 3 | 3 | 3 | 3 | 3 | 5 | 1 | 0.99 | Retained |
| 35 | 3 | 3 | 3 | 3 | 3 | 5 | 1 | 0.99 | Retained |
| 36 | 3 | 3 | 3 | 3 | 3 | 5 | 1 | 0.99 | Retained |
| 37 | 3 | 3 | 3 | 3 | 3 | 5 | 1 | 0.99 | Retained |
| 38 | 3 | 3 | 3 | 3 | 3 | 5 | 1 | 0.99 | Retained |
| 39 | 3 | 3 | 3 | 3 | 3 | 5 | 1 | 0.99 | Retained |
| 40 | 3 | 3 | 3 | 3 | 3 | 5 | 1 | 0.99 | Retained |
| 41 | 3 | 3 | 3 | 3 | 3 | 5 | 1 | 0.99 | Retained |
| 42 | 3 | 3 | 3 | 3 | 3 | 5 | 1 | 0.99 | Retained |
| 43 | 3 | 3 | 3 | 3 | 3 | 5 | 1 | 0.99 | Retained |
| 44 | 3 | 3 | 3 | 3 | 3 | 5 | 1 | 0.99 | Retained |
| 45 | 3 | 3 | 3 | 3 | 3 | 5 | 1 | 0.99 | Retained |
| CVI | | | | | | | 1 | 0.99 | Strong instrument |

***IT-HOME*** Infant-Toddler Home Observation for Measurement of the Environment, CVI Content Validity Index
